# Low-grade appendiceal mucinous neoplasm: A case report

**DOI:** 10.1097/MD.0000000000040911

**Published:** 2024-12-13

**Authors:** Zhitang Guo, Kui Long, Zhanbin Chen, Wei Zhang, Quanxian Chu

**Affiliations:** a Department of Hepatopancreatobiliary Surgery, The Second Affiliated Hospital of Kunming Medical University, Kunming, China; b Department of General Surgery, Nujiang Prefecture People’s Hospital, Nujiang, Yunnan, China.

**Keywords:** case report, low-grade appendiceal mucinous neoplasm, peritoneal pseudomyxoma, surgery

## Abstract

**Rationale:**

Low-grade appendiceal mucinous neoplasm (LAMN) is a clinically rare tumor that predominantly occurs in females and presents with nonspecific symptoms, often resulting in misdiagnosis. While postoperative pathology remains the gold standard for diagnosis, accurate preoperative identification through various diagnostic methods is essential for effective treatment planning. To raise awareness of this condition, we present a case of a middle-aged male diagnosed with LAMN.

**Patient concerns:**

A 52-year-old male presented to outpatient clinic with right lower abdominal pain lasting 1 day. He described the pain as continuous cramping, exacerbated by movement and coughing, with associated nausea.

**Diagnosis:**

Physical examination revealed tenderness in the right lower quadrant and a palpable mass measuring approximately 6.5 cm × 5.0 cm, with poor definition and limited mobility. An elevated white blood cell count (16.2 × 10^9^/L) and a mixed cystic and solid mass were noted, prompting admission for further evaluation. Further, abdominal enhanced computed tomography revealed a mixed-density lesion in the right ileocecal region, measuring approximately 6.5 cm × 5.0 cm. This finding was suggestive of an appendiceal mucinous neoplasm, with mucinous adenocarcinoma remaining a possibility that could not be excluded.

**Interventions:**

The patient underwent a laparoscopic right hemicolectomy on June 5, 2024, and the gross specimen showed: a 6.5 cm × 5.0 cm mass was found in the appendix area on the surface of the intestinal tube, a large amount of jelly was found after incision, and the mass was connected to the intestinal cavity.

**Outcomes:**

The patient recovered well after surgery, the abdominal drainage tube was pulled out on the 8th day after surgery, and the patient was discharged on the 13th day after surgery. Postoperative examination showed LAMN and mucus accumulation in the wall of the appendix with a foreign body giant cell reaction, acute attack of chronic appendicitis, and suppurative inflammation with peripheral inflammation. Postoperative diagnosis: LAMN; acute chronic appendicitis attack.

**Lessons:**

As a rare clinical gastrointestinal tumor, LAMN lacks specific clinical manifestations, and its diagnosis depends on postoperative examination; however, the indications for surgery are clear and the clinical prognosis is good. The key to surgery is to protect the tumor body to avoid rupture and cause the development of peritoneal pseudomyxoma (PMP).

## 1. Introduction

Abdominal pain is regarded as a symptom rather than a distinct pathology,^[1]^ as it can result from a wide range of underlying causes, both known and unknown.^[2,3]^ Among these, acute abdominal pain is 1 of the most frequently encountered presentations in outpatient clinical settings, potentially signaling a spectrum of conditions ranging from benign to life-threatening.^[2]^ Although abdominal pain itself lacks diagnostic specificity, a comprehensive evaluation that integrates the patient’s medical history, physical examination, laboratory findings, and, when indicated, diagnostic imaging can significantly enhance the accuracy of differential diagnoses and guide appropriate therapeutic interventions.^[1,2,4]^ This multidisciplinary approach is crucial for timely and accurate diagnosis, especially in complex or ambiguous cases.

Appendiceal mucinous neoplasms are rare, noninvasive tumors, comprising approximately 0.2% to 0.3% of appendectomy specimens.^[5]^ They encompass a spectrum of pathologies, including low-grade appendiceal mucinous neoplasm (LAMN), mucinous adenoma, and appendiceal adenocarcinoma.^[6]^ Clinically, these neoplasms may present with nonspecific systemic symptoms, abdominal pain, or a palpable mass.^[7–9]^ Additionally, these neoplasms typically manifest in the sixth decade of life, with a higher prevalence in females, while data on male patients remain limited.^[9]^

LAMN poses a significant risk for the development of pseudomyxoma peritonei (PMP), a condition characterized by the accumulation of large volumes of mucin in the abdominal cavity.^[10,11]^ Timely surgical intervention is essential not only to mitigate the risk of PMP but also to enhance long-term patient outcomes.^[12]^ The early recognition and appropriate management of LAMN are crucial for preventing complications associated with this neoplasm. Given the nonspecific nature of its clinical presentation and the potential for misdiagnosis, a multidisciplinary approach is essential to enhance diagnostic accuracy and ensure timely intervention. In this report, we present a case of LAMN in a middle-aged male, aiming to contribute to the growing body of literature and enhance our understanding of this rare entity.

## 2. Case presentation

The patient, a 52-year-old male, presented to the general surgery outpatient clinic at Nujiang People’s Hospital with a complaint of right lower abdominal pain lasting for 1 day. He described the pain as continuous cramping, exacerbated by coughing and movement, with no known relieving factors. The pain was accompanied by nausea but without vomiting, chills, or fever. On physical examination, there was tenderness in the right lower quadrant, with a palpable mass measuring approximately 6.5 cm × 5.0 cm, of moderate consistency and poorly defined borders, with limited mobility. The psoas sign was negative, but the obturator sign and colonic inflation sign were positive. Abdominal ultrasound revealed a mixed cystic and solid mass in the right lower quadrant, with poor sound transmission, suggesting an inflammatory or mucinous neoplasm. Blood analysis showed elevated white blood cell count (16.2 × 10^9^/L), indicating a potential infection or inflammatory process. Given the clinical presentation of a palpable mass, signs of localized inflammation, and the suspicion of a neoplasm based on ultrasound, the patient was admitted for further evaluation. An enhanced abdominal computed tomography (CT) scan was performed after admission to better assess the nature of the mass and to differentiate between possible conditions, such as appendicitis, inflammatory mass, or appendiceal mucinous neoplasm.

**Figure 1.**
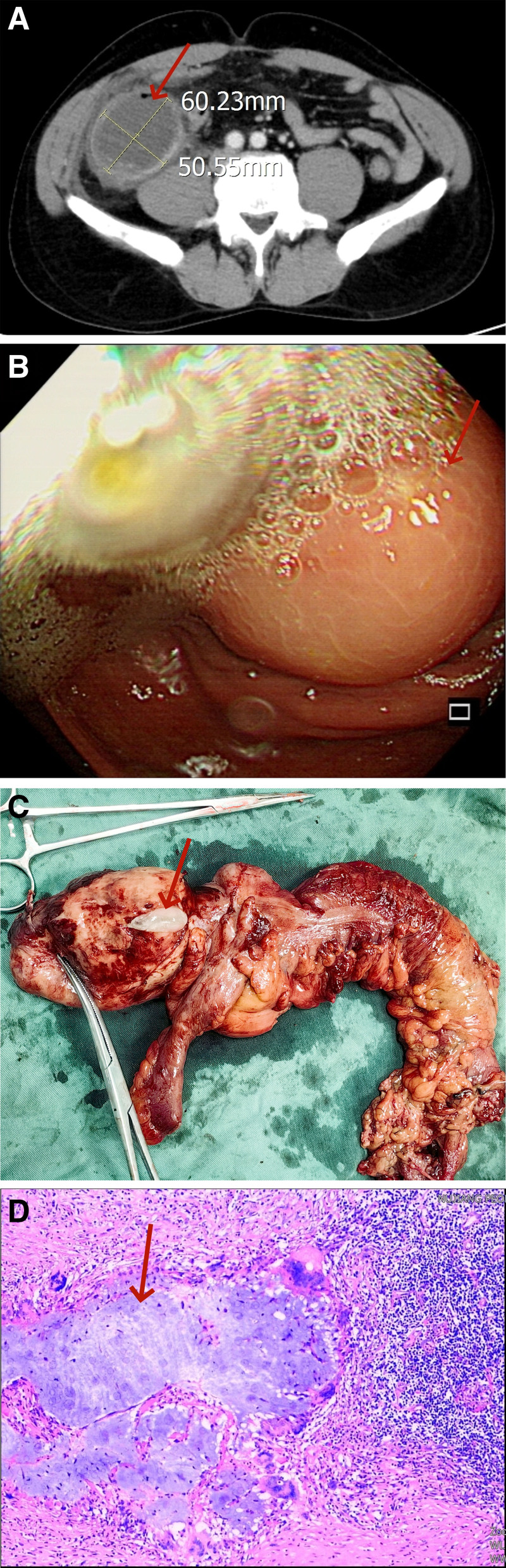
(A) CT examination showed a mass in the right ileocecum. (B) Colonoscopy revealed a tumor in ileocecum. (C) Gross specimen: a large number of jelly in the tumor. (D) Postoperative pathological examination: low-grade appendiceal mucinous neoplasm. CT = computed tomography.

After the enhanced abdominal CT examination, a mixed-density shadow measuring 6.5 cm × 5.0 cm was identified in the right ileocecal region. The mass had a well-defined capsule with scattered calcifications in the wall and areas of liquid density, which showed enhancement on contrast imaging (Fig. 1A). This was suggestive of an appendiceal mucinous neoplasm, and mucinous adenocarcinoma could not be excluded. Colonoscopy revealed a spherical bulge in the ileocecum, approximately 2.5 cm × 2.2 cm in size, with a smooth surface; the mass extended around the periphery of the cecum with attached pus (Fig. 1B). Observation of the base was limited, and entry into the terminal ileum was unsuccessful. Tumor marker testing revealed an elevated CA72-4 level (>300.0 U/mL), while other tumor markers, as well as liver, kidney, and coagulation functions, were within normal limits.

On June 5, 2024, the patient underwent a laparoscopic right hemicolectomy. The gross specimen revealed a 6.5 cm × 5.0 cm mass in the appendix region on the surface of the intestinal tract. Upon incision, a large amount of mucinous material was found, and the mass was connected to the intestinal lumen (Fig. 1C). The patient had an uneventful recovery, with the abdominal drainage tube removed on postoperative day 8, and was discharged on day 13.

Postoperative pathology confirmed a diagnosis of LAMN, with mucinous accumulation in the appendix wall, foreign body giant cell reaction, acute exacerbation of chronic appendicitis, and suppurative inflammation with surrounding tissue involvement (Fig. 1D). Immunohistochemistry showed: CK7–, CK20+, Villin+, CDX-2+, SATB-2+, TTF-1–, P53 weakly positive in 30% of cells, and Ki-67 positive in 70% of cells. The final postoperative diagnoses were: LAMN; acute exacerbation of chronic appendicitis.

## 3. Discussion

LAMN is a noninvasive epithelial tumor characterized by the continuous secretion and accumulation of gelatinous mucin.^[13]^ A significant clinical concern with LAMN is its potential to progress into PMP, a condition marked by the extensive accumulation of mucin within the abdominal cavity.^[10,11,14]^ If left untreated, PMP can lead to organ compression and dysfunction, severely affecting the patient’s quality of life. Therefore, early diagnosis and surgical management of LAMN are critical for reducing the risk of PMP. Research indicates that timely surgical intervention not only effectively lowers the incidence of PMP but also markedly improves long-term patient outcomes.^[12]^

To enhance the accuracy of the differential diagnosis, we employed a multidisciplinary approach that integrated a step-by-step diagnostic process, combining the patient’s medical history, physical examination, laboratory test results, and diagnostic imaging findings. In this case, a 52-year-old male presented with a 1-day history of continuous cramping pain in the right lower abdomen, exacerbated by movement and coughing, accompanied by nausea, but without vomiting, chills, or fever. Physical examination revealed tenderness and a poorly defined, moderately firm mass measuring 6.5 cm × 5.0 cm in the right lower quadrant. Subsequently, abdominal ultrasound showed a mixed cystic-solid mass, raising suspicion for an inflammatory or mucinous neoplasm. Blood tests indicated elevated white blood cell counts (16.2 × 10^9^/L), suggesting infection or inflammation. The patient was then admitted for further evaluation, and an enhanced abdominal CT scan was performed to differentiate between appendicitis, inflammatory mass, or mucinous neoplasm. The CT scan revealed a 6.5 cm × 5.0 cm mixed-density mass in the right ileocecal region, characterized by a well-defined capsule, wall calcifications, and areas of enhanced liquid density, indicating an appendiceal mucinous neoplasm, although mucinous adenocarcinoma could not be excluded. Similar studies have reported comparable diagnostic strategies; however, they often lack comprehensive summaries and clinical recommendations.^[8,9]^ Furthermore, existing literature underscores the importance of diagnostic imaging in the evaluation of acute abdominal pain in adults, emphasizing its role in clinical decision-making.^[2]^ Although the gold standard for diagnosing LAMN relies on postoperative pathology, our multidisciplinary approach strives to achieve the most accurate diagnosis possible, enabling the development of a more tailored treatment strategy. This method underscores the significance of a thorough, step-by-step evaluation, wherein various diagnostic modalities validate and complement 1 another, allowing us to meticulously unravel the complexities of the diagnosis and ultimately enhance patient outcomes.

Due to the well-differentiated nature of LAMN, surgical intervention aims to achieve complete resection with negative margins. Currently, there is no consensus on the necessity for specific follow-up treatment; however, it is essential to exclude the possibility of mucinous adenocarcinoma. If malignant disease is suspected, a right hemicolectomy is indicated. In cases where the tumor involves the mesenteric tissue or the base of the appendix, total resection, including ileocecal resection, should be performed.^[7,15]^ Furthermore, intraperitoneal chemohyperthermia may be warranted if there is evidence of appendiceal perforation or mucinous ascites.^[16]^ In the presented case, a laparoscopic right hemicolectomy was performed because the preoperative assessment could not definitively exclude mucinous adenocarcinoma. Should postoperative examination reveal malignant characteristics, comprehensive adjuvant therapy and close follow-up would be necessary to ensure optimal patient outcomes.

Additionally, LAMN has a higher prevalence in females, while data on male patients remain limited.^[9]^ Given the low incidence of this condition, there is little understanding of whether significant symptom differences exist between genders. For instance, a case report by Mannarini described a 64-year-old female patient with low back pain as the primary symptom of LAMN. In contrast, our case report involved a 53-year-old male patient presenting with acute abdominal pain due to LAMN. Due to the limited data available, it is still premature to draw definitive conclusions regarding potential gender differences in presenting symptoms. However, this observation highlights an important area for future research and clinical attention.

LAMN is a rare gastrointestinal tumor often presenting with nonspecific symptoms. A multidisciplinary approach that integrates a step-by-step diagnostic process is essential for clarifying surgical indications and achieving favorable outcomes. Key surgical strategies focus on preserving tumor integrity to prevent rupture and mitigate the risk of PMP. Furthermore, the differing symptom profiles in male and female patients warrant further investigation to enhance understanding and management of this neoplasm.

## Author contributions

**Conceptualization:** Zhitang Guo, Kui Long, Zhanbin Chen.

**Data curation:** Zhitang Guo, Kui Long, Zhanbin Chen.

**Formal analysis:** Zhitang Guo, Kui Long, Zhanbin Chen.

**Funding acquisition:** Zhitang Guo.

**Investigation:** Zhitang Guo, Kui Long.

**Methodology:** Zhitang Guo, Kui Long, Zhanbin Chen.

**Project administration:** Zhitang Guo, Wei Zhang, Quanxian Chu.

**Resources:** Wei Zhang, Quanxian Chu.

**Software:** Wei Zhang.

**Writing – original draft:** Zhitang Guo.

**Writing – review & editing:** Zhitang Guo, Kui Long.
